# The effects of early marriages on academic performance of marginalized girls in secondary schools of central province in Zambia

**DOI:** 10.1186/s12889-025-24089-x

**Published:** 2025-08-16

**Authors:** Sikota Sharper, Chikumbe Sankwa Evans, Mufalali Simasiku Mwiya, Mwila Bowa, Enock Mutengo, Kwame Nkrumah University

**Affiliations:** Department of Economics and Finance, Kwame Nkrumah University, Plot 1583 Munkoyo Street, Kabwe, Zambia

**Keywords:** Zambia, Education, Female students, Academic performance, Early marriage

## Abstract

Zambia’s education sector faces significant challenges, particularly for female students who are affected by poverty, cultural attitudes, and early marriage. Despite efforts to improve educational access, many girls struggle with enrollment and academic performance. This study aimed to analyze the effects of early marriages on the academic performance of marginalized girls in Secondary Schools in selected secondary schools in the Central Province of Zambia. Adopting a phenomenological approach, the study conducted focus group discussions and interviews with key stakeholders, including marginalized girls, out-of-school girls, school staff, and community leaders. Thematic analysis of data from structured field notes and NVivo software identified six themes including direct costs, inadequate parental support, restrictive gender norms, inclusivity, improving child safety, and dissolving early marriages. The findings indicated that sponsorship initiatives help reduce dropout rates, increase access to education for girls, and improve child safety in schools and communities. Nonetheless, challenges such as poverty, cultural attitudes, inadequate parental support, early marriage, and restrictive gender norms are prevalent. The intricate interplay of poverty, socio-cultural norms, and enduring gender inequalities poses a significant barrier to educational attainment for marginalized girls. Financial constraints and poverty forces families to prioritize survival over education, leaving girls susceptible to early marriage, school dropout, and risky behaviors as coping mechanisms. In conclusion, early marriage greatly harms the education of marginalized girls by making existing problems worse and putting them at greater risk. Solving this issue needs a full plan that lowers school costs, involves the community, keeps girls safe, supports gender equality in schools, and applies laws that stop early marriage.

**Supplementary Information:**

The online version contains supplementary material available at 10.1186/s12889-025-24089-x.

## Background

According to the World Bank, Zambia has one of the highest poverty rates in the world, with over 60% of the population living below the poverty line [3]. This significantly impacts the education sector, as many students cannot access quality education due to financial constraints. Female students, in particular, face additional barriers to education and being marginalized in society [12]. Marginalized girls are those who have dropped out of school, leaving them isolated, voiceless, and deprived of essential educational and financial support. They frequently come from rural, poverty-stricken areas and are affected by harmful practices such as early child marriage, as well as cultural discrimination. As a result, they face heightened vulnerability to violence, HIV, and poor academic performance.

The Zambian government has made substantial efforts to ensure education is accessible to all, focusing on increasing enrolment rates and improving the quality of education [24]. However, many students still face challenges in accessing quality education. A United Nations report indicates that only 36% of Zambian girls are enrolled in secondary school, compared to 42% of boys [25]. This disparity is due to factors such as poverty, cultural attitudes towards education, and early marriage [7, 36].

Despite the positive impact of sponsorship on academic performance [32], female students in Zambia face several challenges in accessing sponsorship [18]. Many families cannot afford the cost of education, affecting the enrolment and academic performance of female students [20, 36]. In some parts of Zambia, girls are expected to marry at a young age, hindering their ability to continue their education [19]. Early marriages also contribute to high rates of school dropouts among female students [17].

In Zambia, 39% of girls are married before the age of 18, and 9% before the age of 15 [11, 39]. Zambia also has some of the highest rates of rural poverty in the world [40]. Within rural Zambia, restrictive gender norms and high levels of poverty make early marriage both a cause and effect of girls’ dropping out of school [17]. According to the United Nations [25], the primary school completion rate for girls in Zambia is lower than that for boys. Additionally, secondary school enrolment rates for girls are lower than for boys [26, 35].

Sponsorship significantly influences academic performance among female students in secondary schools in Zambia [18]. However, female students face several challenges in accessing sponsorship, including restrictive gender norms that prioritize boys’ education, early marriages, limited funding opportunities, and the burden of household chores that hinder their ability to pursue and complete their studies [19]. To address these challenges, increased investment in education, particularly for female students, is necessary. Additionally, sensitization campaigns to promote the importance of education for girls in Zambia are needed. Many sponsors have supported empowering young rural women to transition from school to economic independence, helping vulnerable girls stay in school, learn, and escape early marriage [10, 27].

These sponsorship activities have increased awareness of rights and positive life choices among boys and girls, contributing to an improved understanding and respect for girls’ rights [9]. They have built awareness of children’s rights, driven the creation of by-laws, and supported the implementation of the Re-entry Policy. This has also increased opportunities for further education and employment for these young women [9, 38].

Bursaries and material support implemented by NGOs address the underlying causes of early marriages, such as poverty and restrictive gender norms, through psychosocial, financial, educational, and local governance/community support [18, 24]. However, persistent challenges include the direct costs of education, unsafe living arrangements, distance to school, retrogressive traditional and cultural beliefs, cross-border transactions for early marriage and child labor, and challenges with securing childcare after re-entry. These challenges indirectly affect attendance, retention, and completion rates for girls in secondary school [18, 20, 36].

Educating girls is one of the most effective strategies to combat early marriages, especially as they move to secondary schools. Girls in secondary school are less likely to marry early, as most look forward to finishing school [7, 12]. Hence, this study intends to analyze the effects of early marriages on the academic performance of girls in selected secondary schools. They become aware of unwanted pregnancies and the dangers of premarital sex. Having a mother who has attended secondary education reduces the risk of child death [28]. Obwari [31] notes that in most schools, many bright and deserving students do not access bursaries and some of those who benefit from it still drop out of school for lack of consistency in the allocation of bursaries [31]. Sponsorship allows girls access to quality education and healthcare, enabling them to speak for themselves and stand up for their rights [18].

The lack of school fees and other educational resources has prompted the government and its partners to provide assistance alleviate early marriages [12, 24]. However, it is not yet established whether this sponsorship is yielding good performance among these girls. Furthermore, there is a need to understand the psychological effect it has on these pupils.

## Aim and design

This study aimed to analyse the effects of early marriages on the academic performance of marginalized girls in Secondary Schools in selected secondary schools in the Central Province of Zambia. It employed a phenomenological approach, which is well-suited for building on findings from previous studies. This approach is appropriate as it helps develop concepts that aid in understanding natural phenomena, with an emphasis on the meaning, experiences, and views of participants [1]. By exploring the lived experiences, behaviour, feelings, and perspectives of participants through focus group discussions, this method captures the core aspects of participants’ lives [13, 15]. This exploratory study aims to provide a deeper understanding of sensitive topics, and behaviours that are stigmatized by a marginalized population group [8, 30].

### Sample and setting

The sampling for the qualitative interviews was purposive, ensuring the representativeness of all key stakeholders and variation by including a range of stakeholders with different dimensions of interest. The study included girls from rural areas in the Central Province, school drop-out girls, head teachers, provincial and district program officers, and traditional leaders, participating in face-to face focus group discussions (FGDs), semi-structured interviews (SSIs), and key informant interviews (KIIs). In addition to the 20 KIIs, there were 10 FGDs which lasted for 30–40 min with selected female students. Each FGD comprised no more than 8 participants to ensure a diversity of views while making facilitation manageable. The study excluded girls from affluent families and those performing well academically.

### Data analysis

We used thematic approach to analyse qualitative data [4]. All interviews were digitally recorded and transcribed verbatim. The authors familiarized themselves with the data through repeated reading, noting initial analytical ideas and dividing the transcripts into meaningful units. Each unit was coded, grouped into sub-categories, and ultimately organized into major themes [5]. We also applied the structured notes methodology, where a note-taker summarized key responses during the interview. The second step involved reviewing the structured notes to ensure they were accurately coded. The coding was then compared, and areas of divergence were noted, discussed, and appropriate actions were taken. The sample size was deemed sufficient based on repeating themes and sub-themes throughout the data, further data collection didn’t yield new insights.

### Trustworthiness

This research was guided by four trustworthiness criteria including credibility, transferability, dependability, and confirmability and Consolidated Criteria for Reporting Qualitative Research (COREQ) checklist. Credibility was secured through robust research methods, supervision by senior researchers, and a clear discussion of how the investigator’s background could influence findings. Transferability was achieved via comprehensive literature reviews, broader contextualization, and comparisons with similar studies. Dependability was strengthened by rigorous, transparent methods, while confirmability was addressed by defining the study’s rationale, acknowledging possible biases, and documenting limitations. Potential biases included interviewer recall bias and the fact that most participants were girls. Five lecturers facilitated the training and data collection process, yet professional boundaries were maintained, and discussions remained focused on the research questions to minimize bias. Their expertise deepened the inquiry without steering participant responses. Overall, the study balanced sound methodology with reflexivity. Ongoing researcher reflection and COREQ checklist supported its authenticity and reliability, demonstrating a commitment to thorough investigation and self-awareness.

### Ethical approval

We obtained ethical approval from Kwame Nkrumah University Ethics Committees (reference number KNU/2023REC04/001). All study personnel were trained in human subjects research ethics before study initiation. The team adhered Belmont Report (1979) to ethical standards of respect for Persons, beneficence and justice. The team exercised the highest degree of professionalism, impartiality, and objectivity at all times. The team secured the informed consent of participants prior to all data collection activities and ensured that interviews and focus group discussions are undertaken in a manner that pays due respect to cultural practices and beliefs. Privacy, the anonymity of participants, and the confidentiality of information was ensured throughout the study. All study participants were provided with participant information sheets detailing the study’s rationale and objectives.

## Results

### Demographics

A total of 71 girls participated in FGDs in their respective schools, along with 5 head teachers, 3 provincial and district program officers, 5 traditional leaders, and 8 purposefully selected parents of out-of-school girls. The girls’ ages ranged from 12 to 22 years, while teachers ranged from 27 to 61 years old, of whom 3 were women. Based on all participants’ insights, the data were organized into themes and sub-themes, as shown in Table 1. The Key Informant Interview (KII) guide utilized in this study is provided as a supplementary file.

**Table 1:** Theme and sub-themes

Main theme	Sub-theme
Direct Cost	-Long distances between home and school -High costs (tuition, uniforms, books) -Inadequate boarding facilities
Inadequate Parental Support	-Lack of financial resources and parental guidance -Need to seek piecework or income generating activities -Early marriages and pregnancies leading to dropout
Restrictive Gender Norms	-Patriarchal social structures devaluing girls’ education -Preference for marrying off girls due to immediate economic gain
Inclusivity	-Positive discrimination to prioritize girls’ enrolment -Gender sensitive curriculum and teaching practices -Remedial support for low performing students
Improving Child Safety	-Threats posed by long travel distances -Building schools closer to communities -Providing safe boarding and supervision
Dissolving Early Marriages	-Community led child safeguarding policies -Village level protection committees and church involvement

#### Direct costs of attending school and dangers of unsafe out-of-school accommodations

School accessibility is a significant challenge in rural areas of Zambia. Schools are often far apart, with students traveling from distant locations to attend. The costs of school requirements, such as uniforms, books, and tuition fees, are prohibitively expensive for many families. As a result, marginalized girls sometimes fail to attend classes due to the distance and hunger. Boarding facilities in schools are nearly at capacity or full. The long distances contribute to dropouts, as some girls cannot manage to walk more than 10, 15, or even 20 km each day and eventually stop attending without notice.

*“The major challenge I encountered during my time was the long distance to school and the lack of parental support to continue learning and complete school. My parents could not afford to buy me shoes*,* socks*,* books*,* uniforms*,* or pay tuition fees*,* and I would borrow money from friends.” Out-of-school girl*

“Another significant challenge is the lack of supervision and control for girls who can afford to rent boarding houses in the community”- Parent

*“Another challenge is that girls who can afford to rent a boarding house have no supervision in the community. They must fend for themselves*,* search for food*,* and this has led to early pregnancies as many engage in early sex” -Teacher.*

*“Girls resort to having boyfriends to pay for school fees and school requirements*,* parents force them to get married for dowry*,* some parents tell girls to go to bars to look for boys” Student.*

*“Another challenge is that not everyone can afford to attend boarding schools. As a result*,* some students move to villages near the school and rent homes. Typically*,* a group of girls will find a house and live together. In this situation*,* there is little to no supervision. The girls have to fend for themselves*,* manage their own meals*,* and look for food. This lack of oversight often leads to early pregnancies”-marginalized girl.*

#### Economic barriers and inadequate parental support lead to high dropout rates and early marriages

Dropout rates increase in areas where learners are forced into early marriages or leave to seek employment. Pupils often search for piecework, leading to higher dropout rates in some schools. Girls who get pregnant are usually married off after delivery and do not return to school. Some parents leave the province or country for extended periods to order merchandise, leaving their school-going children in charge of the family. Consequently, these children may stop attending school without informing teachers, as they have to manage family businesses and fend for younger siblings.

*“The dropout rate is incredibly high due to early marriages and pregnancies*,* especially in rural setups.” Government official*

*“Many people also believe that if you get pregnant*,* you should eventually get married to the person who impregnated you.” Out-of-school student*

*“The major challenge I encountered during my time was the lack of parental support for me to continue learning and complete school.” School drop-out.*

*“Seasonal activities like caterpillar harvesting during the rainy season*,* prevent girls from attending school regularly. Families rely on this income*,* so even schoolgirls are taken to gather caterpillars.” Parent.*

#### High dropout rates due to cultural attitudes, poverty, and restrictive gender norms

High illiteracy levels contribute to dropout rates by reducing morale and affecting performance and motivation. Some parents in rural areas still have negative attitudes towards girls’ education, preferring the immediate benefits of marrying off their daughters, such as receiving cattle and goats. Continued sensitization is necessary to change these attitudes. Poor nutrition also leads to dropouts, as some children do not have enough food. Additionally, some parents or guardians may be unwilling to share reasons for their children’s dropout, especially if it involves early marriage. Traditional and cultural beliefs also play a role, with child mothers often lacking support to leave their children and return to school [20, 38]

Qualitative interview respondents described Zambia as a patriarchy, where societal norms prioritize boys over girls in many endeavors. Social norms around early marriage devalue girls’ education, particularly in poor households where girls are expected to be caregivers and homemakers. This is amplified by the lack of visible pathways for girls and fewer educated and successful female role models.

*“…being a girl*,* it almost doubles punishment because they have to do the chores at home before school and if they are late to school*,* they get punished”* Head teacher.

*“They encourage gender equality at all costs; everyone is equal and can be what they want to be if they are ambitious.”* Student.

*“Poverty and its consequences lead marginalized girls to become pregnant. The lack of resources to support children both in and school drop-outs is critical*,* prompting some girls to engage in risky behaviors to obtain these resources.” head teacher.*

Poverty is a major underlying cause of early marriage, exacerbated in rural areas where over a third of the population is poor. Parents or guardians of marginalized girls in rural areas often lack additional income and are subsistence farmers. Poverty discourages marginalized girls from accessing education due to direct or indirect costs, leaving them vulnerable to early marriage [12]. In some cases, poverty leads girls to engage in transactional sex with older men who provide for them financially, resulting in early pregnancies and marriages. Some parents or guardians even encourage their daughters to seek sexual encounters as a means of financial support.

####  Inclusivity and equity in schools, increasing access for girls and low-performing students

Most key informants described challenges in prioritizing girls’ education, detailing cultural and social norms that favor girls as caregivers and housekeepers instead of being educated or breadwinners. Key informants also described low access to education for low-performing students, stating that these children often struggle to get placement in schools. Marginalized communities also struggle to access private schools that may provide differentiated learning.

*“Positive discrimination makes girls a priority in everything. Engaging girls in dialogue to address the problems they are facing and equipping girls with knowledge so they can make informed decisions.” Head Teacher.*

The Government of Zambia school bursary program has effectively addressed this challenge by promoting inclusivity and equity in schools. The selection process prioritizes average and low-performing students, often overlooked by other schools. The school’s gender policy of 50%+1 ensures more girls are enrolled, contributing to closing the gender gap. Initiatives such as girls’ clubs, the provision of sanitary towels, and clothes for the most vulnerable girls, and high engagement with parents and communities on the value of girls’ education have all supported this effort.

*“The schoolteachers treat everyone equally*,* irrespective of their social status*,* and they take care of the girls when they need sanitary towels.” Community member.*

*“We further have a gender-sensitive curriculum that addresses the needs of both genders.” Student.*

Teachers interact well with all learners and treat them equally. Key informants reported that teachers respect all children, regardless of their social status. The gender-sensitive curriculum addresses the needs of students at school, promoting inclusivity and equity.

*“Teachers identify those who do not know how to read and write and make sure they learn by giving them homework. During holidays*,* they introduce radio programs and pair students in groups monitored by teachers.” Teacher.*

Schools also provide additional support for low-performing students, including remedial classes, weekly tests, holiday homework, and radio programs. Students are paired in groups and closely monitored by teachers, receiving more contact time. Significant engagement with parents ensures teachers have adequate insight into each child's situation. Students attend a full day of teaching, unlike the half-day provided in other schools due to limited facilities. Tertiary education bursaries incentivize students to improve their performance.lso provide additional support for low-performing students, including remedial classes, weekly tests, holiday homework, and radio programs. Students are paired in groups and closely monitored by teachers, receiving more contact time. Significant engagement with parents ensures teachers have adequate insight into each child's situation. Students attend a full day of teaching, unlike the half-day provided in other schools due to limited facilities. Tertiary education bursaries incentivize students to improve their performance.

#### Child safety in schools and the community

Long travel distances increase the likelihood of abuse or transactional sex for marginalized girls. Many children lack adequate resources to meet their school and domestic needs. Most girls have limited exposure beyond their villages, making them easily influenced and turning to sexual activity to meet their needs. Day students are more affected than boarders, who are better supervised. Some out-of-school boarding houses are not well monitored.

*“As girls go to school and walk long distances*,* they are approached by truck drivers or random men who entice them with money or rape them.” Parent.*

The distances children travel to school in rural communities are not only physically straining and economically challenging but also a safety concern. Students have been harmed in the past during these trips, leading to fear and refusal to attend school. The construction initiative, building schools close to homes and providing boarding facilities, has addressed these security issues. Boarding facilities enhance the protection of children, especially girls, allowing them to focus on academics without the added pressure of household chores or distractions from male advances.

#### Dissolving early marriages, returning girls to school, and supporting the care of babies post-re-entry

Over 80% of key informants described cultural practices that increase curiosity and promote early sexual debut, as well as societal pressure for girls to have children early. Educating girls is sometimes seen as a waste of resources, especially since there are economic gains from marrying them off. Societal and religious norms disapprove of unwed mothers, motivating parents to marry off their daughters early to avoid pregnancy outside of wedlock or community ridicule.

*“Girls drop out of school because of early pregnancies and early marriages*,* and the family responsible often does not take the girl back to school after pregnancy.” Parent.*

*“Village leaders meet with the school management to address school affairs. Each village has a committee on child protection policy and reports vices like early marriages and pregnancies. The school works with churches to teach good values to children.” Community leader.*

Early marriage prevents enrollment or causes girls to drop out of school. Most parents do not support girls returning to school after childbirth, with responsibilities shifting to the baby’s father’s family. However, the father’s family often does not support the girl’s re-entry.

*“Child protection emphasizes many things*,* and it was our job to meet with parents and make an executive in every village to look at child protection. Anyone keeping a child at home was reported*,* and the child returned to school after explaining the reasons.” Traditional leader.*

The school program’s child safeguarding policy, involving extensive community engagement, has been effective. Community sensitization efforts have involved parents, traditional leaders, community leaders, and the school, raising awareness of the negative impact of early marriages on education. Village-level child protection committees report early marriages and pregnancies. The village executive collaborates with the school and parents to reintegrate girls into the community and school. Partnering with community leaders and churches has built a system that dissolves early marriages, returns girls to school, and supports babies’ care post-re-entry.

## Discussion

Early marriage is a deeply rooted social problem with significant implications for the education of girls in rural areas [36]. Despite efforts to improve girls’ access to education, numerous barriers remain, particularly in rural regions where poverty and traditional cultural practices are prevalent [12, 22, 35]. This qualitative research study aimed to examine the impact of early marriages on the academic performance of marginalized girls in secondary schools in Central Province, Zambia.

The findings revealed that one of the primary challenges faced by girls in rural areas regarding education is the direct costs associated with schooling. High expenses related to attending school, such as purchasing uniforms, and books, and paying tuition fees, act as significant obstacles for these girls, most of whom come from impoverished households. In these contexts, girls are often seen as a financial burden. Furthermore, inadequate boarding facilities in schools force some girls to seek accommodation in the community, exposing them to unsafe environments and increasing their vulnerability to early pregnancies. This aligns with the findings of [33], who established that high education costs can contribute to early marriages in rural communities, as some parents opt to marry off their daughters instead of sending them to school. Both the direct and indirect costs associated with schooling, along with long travel distances and lack of safe accommodation, pose significant barriers to school attendance and retention [2, 34].

Moreover, the study indicates that pregnancy and early marriage are major factors contributing to high dropout rates among marginalized girls in the region. Many girls are compelled to leave school due to pregnancy and are often married off to the men who impregnated them, with little support from their parents or the community to continue their studies. The lack of parental support, both financially and emotionally, further exacerbates the challenges these girls face in completing their secondary education. A study by Sommer [37] highlights the negative impact of reluctant parental support for girls’ education, attributing it to the preference for educating sons when limited funds are available for school fees, which increases the pressure on girls to marry early and may lead to premarital pregnancies.

Furthermore, the research emphasizes the role of illiteracy and negative attitudes toward girls’ education in rural areas as factors contributing to high dropout rates. Some parents in these communities still consider girls’ education less important than their traditional roles as caregivers and homemakers [16], prioritizing early marriage over continued schooling. This finding is supported by other researchers such as [21, 34] who conclude that societal perceptions of girls’ roles, cultural pressures, and the perceived value of girls as future mothers and homemakers have detrimental consequences for girls’ education.

The findings also identify poverty as a major underlying cause of the challenges faced by marginalized girls in accessing and completing secondary education. Financial constraints discourage families from investing in their daughters’ education, leaving them vulnerable to early marriage and pregnancy as a means of survival. Financial hardships in poor households force many children, especially girls, *drop-outs*, sometimes leading them to engage in transactional sex, further increasing their risk of early pregnancy and dropout [14, 29].

However, progress has been made in addressing these issues through various interventions [26]. Programs such as the re-entry policy have been implemented in schools, allowing pregnant girls and young mothers to return to their studies after childbirth [6]. Schools collaborate with parents and extended families to provide support and prevent stigmatization, ensuring successful reintegration for girls [23]. Some girls who have experienced this process have become counselors to support others in similar situations.

Additionally, the establishment of school boarding facilities aims to provide a protected environment for girls, shielding them from risks such as sexual exploitation and abuse. Community engagement efforts, including working with village leaders and child protection committees, have helped address issues like early marriage and early pregnancy. Nyamanhindi in 2013 recognized the effectiveness of such engagements in educating the community about harmful norms and practices, thereby promoting inclusive and equitable education for both boys and girls and upholding education as a universal human right [14, 29].

Furthermore, the government’s efforts to build schools closer to rural communities have reduced the long and unsafe distances that students have to travel. The provision of free education, learning materials, and other support structures has been well received by the community and teachers, helping eliminate financial barriers for marginalized girls and the general community. Remedial classes, regular testing, and personalized attention from teachers are employed to support underperforming students. Additionally, incentives like tertiary education bursaries have been introduced to motivate students to improve their academic performance, facilitating their entry into universities and colleges where such incentives are offered [18, 24].

Despite these positive developments, the study underscores the ongoing need to address the persistent challenges faced by marginalized girls in accessing and completing their education. Sustainable solutions must adopt a comprehensive approach that addresses the intersecting factors of poverty, socio-cultural norms, and gender inequalities

## Limitations

The study acknowledged that participants who had experienced early marriage might find discussing these experiences distressing. The research team approached all participants with sensitivity during recruitment, emphasizing that they were not obligated to disclose any sensitive or emotionally charged information. Rather, participants were free to share only what they felt comfortable discussing. Due to the sensitive nature of the topic, certain details may not have been captured, potentially affecting the rigor of the findings. Moreover, the study was limited in scope and did not comprehensively address all facets of early marriage.

## Conclusion

The discourse on early marriage and its impact on girls’ education in rural areas resonates deeply with the ongoing struggle for gender equality and human rights, not just in the Kabwe District of Zambia but also around the world. The findings in this research, along with the scholarly literature presented, unveil the intricate web of challenges that impede marginalized girls’ access to education.

It has been established that the complex interplay of poverty, socio-cultural norms, and persistent gender inequalities has created formidable barriers to educational attainment for these girls. The research underscores how illiteracy and negative attitudes toward girls’ education in rural areas have substantially perpetuated gender disparities and hindered the educational advancement of girls. Financial constraints compel families to prioritize survival over education, leaving girls vulnerable to early marriage and pregnancy as coping mechanisms. These financial hardships further exacerbate girls’ risk of dropping out and engaging in risky behaviors, worsening their potential educational outcomes.

Despite glimpses of progress through targeted interventions and community-driven initiatives aimed at addressing root causes and fostering inclusive educational solutions such as the re-entry policy and free education. Stakeholders, including scholars, practitioners in the Ministry of Education, policymakers, and governments, must heed the insights gleaned from this study. They must advocate for more practical interventions that prioritize gender equality and the right to education for all.

The research underscores the urgency of adopting a comprehensive, context-sensitive approach that places the unique needs and experiences of marginalized girls at the center of educational reform efforts in rural areas of Zambia. Accordingly, based on the findings, we recommend the following:

- Recommendations
  1. **Expand and strengthen bursary programs in rural areas.** Increase the scope of existing bursaries and scholarships to cover tuition, uniforms, books, and other school-related costs, ensuring that financial constraints do not force girls into early marriage or push them out of school. Governmental and non-governmental organizations introduce income-generating activities for families in rural areas. This approach will ease the financial burden that often drives early marriage and school dropouts.
  2. **Improve boarding facilities and enforce re-entry policies in rural areas.** Provide safe, affordable boarding options for girls who live far from schools, minimizing the risks associated with unregulated accommodation. At the same time, ensure that re-entry policies for pregnant and married girls are effectively implemented, offering counselling, childcare support, and ongoing mentorship.
  3. **Increase community sensitization and child protection mechanisms. **Collaborate with traditional leaders, churches, and community groups to challenge harmful norms about girls’ education and highlight its long-term benefits. Reinforce local child protection committees that can rapidly address early marriages, pregnancies, or abuse, and coordinate efforts to bring affected girls back to school.
  4. **Promote parental engagement and peer mentorship in rural areas.** Engage parents in regular meetings and discussions through village headmen and chiefs, emphasizing the value of girls’ education and strategies for financial planning. At the same time, foster peer mentorship programs where successful female students or graduates guide and support at-risk girls, offering motivation and real-life success stories.
  5. **Adopt gender-sensitive teaching in schools.** Train teachers in gender-responsive pedagogies and ensure curricula highlight female role models to inspire students. Implement a strong monitoring and evaluation system to track enrolment, dropout, and re-entry rates using school data

## Supplementary Information

Supplementary Material 1.

Supplementary Material 2.

Supplementary Material 3.

## Data Availability

The data and transcripts that support the findings of this study are available from the corresponding author upon request.
