# Supplementary material for: The effects of early marriages on academic performance of marginalized girls in secondary schools of central province in Zambia
Source: BMC Public Health. 2025 Aug 16;25:2815. doi: 10.1186/s12889-025-24089-x (PMC12357362; doi:10.1186/s12889-025-24089-x)
Supplement: Supplementary file 1 — Supplementary Material 1. [file 12889_2025_24089_MOESM1_ESM.pdf]

KWAME NKRUMAH UNIVERSITY

DIRECTORATE OF RESEARCH, POSTGRADUATE STUDIES AND INNOVATION

P.O. BOX 80404, KABWE, ZAMBIA
Website: http://www.nkrumah.edu.zm
PHONE: 0973780447/0973650600
TEL/FAX: 021 5-23223

ETHICS CERTIFICATE OF APPROVAL

Certificate Reference Number: KNU/2023REC04/001

Project title: **The effects of child marriages on academic performance of marginalized girls in Secondary Schools of Central Province in Zambia..**

Principal Researcher: Mr Sharper Sikota

Protocol No: Project 2022REC09/001: Dated: 02-April-2023

Participant Information and Consent Form. Dated: 02-April-2023

On behalf of the University Research Committee (UREC), I hereby give ethical approval in respect of the undertakings contained in the above-mentioned project and research protocol(s) as considered by the committee on 12th April 2023. You are advised to take note of the conditions under which this approval is granted as provided. Should any other instruments be used, these require separate authorization. You may therefore commence with the research as from the date of this certificate, using the reference number indicated above.

Please note that the UREC must be informed immediately of

1. Any material changes in the conditions or undertakings mentioned in the application form document
2. Any material breaches of ethical undertakings or events that impact upon the ethical conduct of the research
3. Any significant change to the project and the reason for that change, including an indication of ethical implications (if any);
4. Serious adverse effects on participants and the action taken to address those effects;
5. Any other unforeseen events or unexpected developments that merit notification;
6. The inability of the Principal Researcher to continue in that role, or any other change in research personnel involved in the project;
7. A delay of more than 12 months in the commencement of the project; and,
8. Termination or closure of the project.

The UREC retains the right to

1. Withdraw or amend this Ethical Clearance Certificate if
   - Any unethical practices are revealed or suspected
   - Relevant information has been withheld or misrepresented
   - Regulatory changes of whatsoever nature so require
   - The conditions contained in the Certificate have not been adhered to

2. Request access to any information or data at any time during the course or after completion of the project.

All research subject to the Kwame Nkrumah University Research Ethics Committee review must be conducted in accordance with the university's Research Ethics Policy.

Kindly note that the Research Committee may conduct an audit at any time.

The Research Committee wishes you well in your research.

Yours sincerely

Dr. Patrick Sibanda
Director – Research, Postgraduate Studies and Innovation

12 April, 2022
