# Supplementary material for: The effects of early marriages on academic performance of marginalized girls in secondary schools of central province in Zambia
Source: BMC Public Health. 2025 Aug 16;25:2815. doi: 10.1186/s12889-025-24089-x (PMC12357362; doi:10.1186/s12889-025-24089-x)
Supplement: Supplementary file 2 — Supplementary Material 2. [file 12889_2025_24089_MOESM2_ESM.pdf]

KIIs Guide

General Information: All participants should be provided with a short introduction to the evaluation prior to the interview/discussion. Facilitators will emphasize specific questions based on interviewees role. Participants will also be informed that the interview will be recorded for quality assurance and analysis purposes only and will be deleted subsequently.

Introduction

What activities have done around ending child marriages in your Chiefdom/Village?

Lived Reality of Marginalized Girls

1. What do you feel are the key challenges in your district for marginalized girls' in relation to their enrolment, attendance, retention, dropout rates and completion rates, early marriage, and early pregnancies, etc. **Probes:** Range of barriers to girls participation within this district, Prevalence of early/child marriage and teenage pregnancy, Impact of distance to school on attendance and retention.

2. What is the level of awareness and promotion among your subjects of children's rights regarding early marriage?

3. How does your community view early marriage, what is acceptable, what is not acceptable? **Probe:** Cultural beliefs and social norms around early marriage, age considered acceptable in this community for girls to get married.

4. How prevalent is dropping out of school because of pregnancy and/or early marriage? **Probe:** Who decides whether a girl should drop out of school to get married? Why?

5. How does the community view the roles of males and females (gender)? How does this contribute to early marriage?

6. Why do parents marry off their daughters early? To what extent is early marriage seen as the best available option, or as a coping strategy by struggling families to relieve their economic burden?

7. What is the impact of COVID-19 on school going children in your community? ***Probe:*** *access to education at home, Perceptions on learning during lockdown, girls' perceptions of risk of dropping out of school.*

8. What do marginalized girls need most during the pandemic to facilitate access to education and learning?

9. What role have you played over the past three years in supporting and enabling girls to attend, remain in school and complete secondary school? I.e. preventing early marriage and dropout. *Probe: Support systems for girls to attend, progress and complete secondary school*

10. To what extent do you feel the project interventions led to a reduction in girls dropping out of school due to pregnancy and/or child marriage? Can you give specific examples to support your answers? ***Probe****: Impact of* ***bursaries*** *on attendance, retention and reduction in dropout rates.*

Local Policy Advocacy and Enforcement

1. What is being done by local government and traditional authorities and civil society structures to tackle the problem of child marriage in your communities? ***Probe*** *on policy enforcement*

2. To what extent do you think schools actively promote the re-entry policy? ***Probe:*** *What do you consider "active" promotion of REP? What steps are schools taking to actively promote the Re-entry Policy?*

3. What do schools in your community encourage girls to return to school after giving birth? ***Probe:*** *How are these young mothers supported*?

4. What other support do you feel girls need to enable them to attend, progress and achieve well in school?

5. What do you perceive as effective strategies and solutions that could prevent girls from marrying early? *Probe: Why?*

6. What is being done by local government and traditional authorities and civil society structures to tackle the problem of child marriage in your communities?

Cross-cutting

1. What role has the school undertaken to support the project interventions in creating a safer, more enabling learning environment particularly for girls?
(including in-boarding if appropriate) ***Probe****: Impact of child protection mechanisms, systems, and processes for reporting.*

2. Which of the interventions have been most successful in reducing / preventing early marriage? ***Probe:*** *Intervention enabling young women to return to school after pregnancy? Which of these interventions do you feel are sustainable post-project? Why?*

3. What other strategies or solutions, do you perceive as effective to prevent girls from marrying early? *Probe: Why?*

Recommendations

1. What are the key lessons and examples of best practice from this project that can be used to inform and improve future support to marginalized girls?

Thank respondents for their participation
